# Eco-Friendly Method for Tailoring Biocompatible and Antimicrobial Surfaces of Poly-L-Lactic Acid

**DOI:** 10.3390/nano9030428

**Published:** 2019-03-13

**Authors:** Magdalena Aflori, Maria Butnaru, Bianca-Maria Tihauan, Florica Doroftei

**Affiliations:** 1 Petru Poni Institute of Macromolecular Chemistry, 41A Grigore Ghica Voda Alley, Iasi 700487, Romania; mariabutnaru@yahoo.com (M.B.); florica.doroftei@icmpp.ro (F.D.); 2 Sanimed International IMPEX SRL, Sos. Bucuresti—Magurele, nr. 70F, Sector 5, Bucharest 051434, Romania; bianca.tihauan@sanimed.ro

**Keywords:** chitosan, poly-L-lactic acid, plasma, silver nanoparticles, antimicrobial

## Abstract

In this study, a facile, eco-friendly route, in two steps, for obtaining of poly-L-lactic acid/chitosan-silver nanoparticles scaffolds under quiescent conditions was presented. The method consists of plasma treatment and then wet chemical treatment of poly-L-lactic acid (PLLA) films in a chitosan based-silver nanoparticles solution (Cs/AgNp). The changes of the physical and chemical surface proprieties were studied using scanning electron microscopy (SEM), small angle X-Ray scattering (SAXS), Fourier transform infrared spectroscopy (FTIR) and profilometry methods. A certain combination of plasma treatment and chitosan-based silver nanoparticles solution increased the biocompatibility of PLLA films in combination with cell line seeding as well as the antimicrobial activity for gram-positive and gram-negative bacteria. The sample that demonstrated from Energy Dispersive Spectroscopy (EDAX) to have the highest amount of nitrogen and the smallest amount of Ag, proved to have the highest value for cell viability, demonstrating better biocompatibility and very good antimicrobial proprieties.

## 1. Introduction

Nowadays, there is a great demand for obtaining new environmentally-friendly and cost-effective materials to replace plastic products [1,2]. One of the most promising polymers is poly-L-lactic acid (PLLA); however, it has limited practical applications due to its low thermal stability and inherent brittle nature. Reinforcements with different substances prove to be a powerful tool in designing clean, eco-friendly materials for several applications [2]. PLLA is widely used in tissue engineering due to its slow degradation rate (almost 6 months to 1 year for complete degradation) [3,4] and cost-effective and effortless large scale production [5]. In this context, a fairly new area is represented by nanocomposites—the reinforcing material having the dimensions in nanometric scale [6]. Noble metal nanoparticles are of increased interest because of their potential applications in novel technologies due to their different properties compared to bulk metals [7,8]. Recently, silver and gold nanoparticles biosynthesis under eco-friendly conditions by using plant extracts, bio-organisms, proteins, and polysaccharides have gained an increased interest from material science researchers [9,10]. In this context, chitosan is one of the most used biopolymers for such approach mainly due to unique physicochemical properties in the presence of largely free amino and hydroxyl groups [11]. Chitosan is also recommended by proprieties like biocompatibility, non-toxicity, bioadhesivity, biodegradability, safety, and the promotion of drug absorption [12]. PLLA has been blended with chitosan to improve its wettability [13] or its tensile strength [14]. Some authors used chitosan as both capping and reducing agent for the incorporation of silver nanoparticles into the polymer matrices [15,16,17]. Due to the ion–dipole intermolecular forces, chitosan stops the aggregation of silver in clusters at the macroscopic level and has a crucial role in stabilization of the formed nanoparticles [18].

Surface treatment procedures, such as plasma discharges in different kind of gases, can modify the physico-chemical proprieties at a scale of only a few atoms layers thick, without changing the bulk material properties [19,20]. Within the last decade, plasma technique has been applied to improve PLLA surface hydrophilicity, roughness and morphology and to proliferate the selective interaction between polymer surface and proteins. Some authors observed that melt extruded PLLA sheets treated with different kind of inert gas discharges did not affect PLLA biodegradation rate in soil [21]. Other authors used plasma treatment in reactive gases for enhancing cell (human skin fibroblast) adhesion on PLLA by obtaining reactive amine groups and further to immobilize collagen through polar and hydrogen bonding interactions at the treated film surfaces [22,23]. By increasing plasma treatment time, an increase of PLLA film degradation takes place [20]. It is well known that some issues related to non-permanent surface modification can occur, making polymers films unsuitable for certain applications in medicine or package food industry [24].

In this study, a facile, eco-friendly route, in two steps, for obtaining of PLLA/Chitosan-silver nanoparticles nanocomposite scaffolds under quiescent conditions was presented. This scaffold proved to be suitable for cell (preosteoblastic cell line MC3T3-E1 established from mouse C57BL/6 calvaria) attachment and proliferation and to have very good biocide proprieties.

## 2. Material and Methods

### 2.1. Materials

Chitosan flakes (molecular weight 50,000–190,000 Da based on viscosity, 75–85% deacetylated), silver nitrate and acetic acid were purchased from Sigma-Aldrich Chemie GmbH, Steinheim, Germany. Solutions were prepared with MilliQ water. Poly-L-lactic acid films of 50 µm thick were purchased from Goodfellow Cambridge Ltd, Huntingdon, UK and were cut in square shapes of 5 × 5 cm size. 

Culture media and solutions: Alpha Minimum Essential Medium (α MEM, with ribonucleosides, deoxyribonucleosides, 2 mM l-glutamine and 1 mM sodium pyruvate, without ascorbic acid GIBCO, Custom Product, Catalog No. A1049001); Bovine fetal serum (BFS); Penicillin/Streptomycin/Neomycin solution (P/S/N) for cell culture; Phosphate Buffered saline (PBS) for cell culture; 3-(4,5-Dimethyl-2-thiazolyl)-2,5-diphenyl-2H-tetrazolium bromide (MTT), solution in PBS (5 mg/mL) were purchased from Sigma-Aldrich Chemie GmbH, Steinheim, Germany. 

Cells: Preosteoblasts of MC3T3-E1 line, subclone 4 (passage 21) (purchased from Sigma-Aldrich Chemie GmbH, Steinheim, Germany) were thawed and multiplied in culture flasks with a surface of 75 cm^2^, in culture media α MEM, without ascorbic acid, supplemented with 10% BFS and 1% mixture of antibiotic. The initial density of cells culture was 2000 cells/cm^2^ culture surface.

### 2.2. The Two Step Method

#### 2.2.1. In Situ Formation Silver Nanoparticles on Chitosan

**Figure 1:**
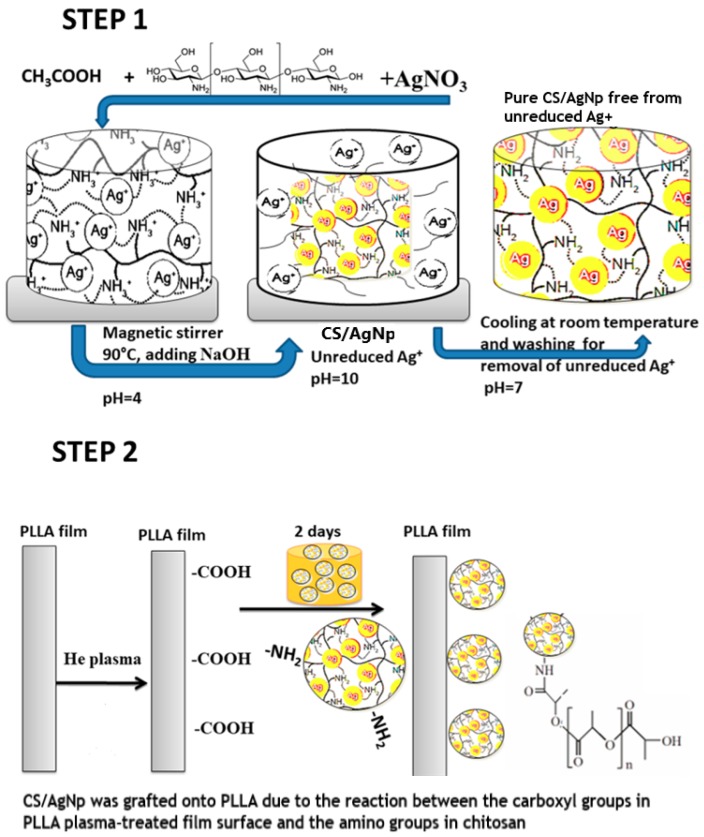
Scheme of two steps experimental method.

Chitosan in a concentration of 6.87 mg/mL was dissolved in 1% glacial acetic acid solution. To avoid nanoparticle sedimentation due to the poor solubility of chitosan, the mixture was kept two days until a clear solution was obtained (Figure 1 Step 1) [25,26]. The solution was prepared by adding 5 mL of 9 mM AgNO_3_ to 10 mL chitosan solution under stirring for 30 min at room temperature. The mixture was transferred to glass tubes and it was kept at 90 °C for 6 h in a temperature controlled bath. After synthesis, the AgNPs colloids were cooled at room temperature for the removal of the majority of the unreduced Ag^+^ to avoid the toxicity of the solution [27,28]. Then, the CS/AgNP solution was stored at room temperature and in dark glass tubes.

#### 2.2.2. Combined Nonconventional and Conventional Treatments of PLLA Films

Briefly, the plasma treatments of PLLA films (Figure 1, Step 2) were performed in an EMITECH RF plasma device. Different times of treatments were performed to obtain a certain concentration of functional groups at the surface layer, without affecting the polymer bulk. The polymer was introduced in a gas vessel containing helium (He) at a pressure p = 5 × 10^–2^ mbar. Right after plasma treatments, to avoid the aging effect, the polymer was immediately immersed in the CS/AgNp solution at room temperature, in a dark place. After 2 days, the samples were rinsed with MilliQ water. Different input RF powers and times were performed for plasma treatments; however, for the present work, the following samples were selected: neat PLLA (P0) film, PLLA films treated in plasma for 4 min at 30 W, immersed in CS/AgNp (P1) and PLLA films treated in plasma for 10 min at 30 W, immersed in CS/AgNp (P2). For a lower time, no significant changes in the surfaces were observed, while for higher times the surface of PLLA film starts the degradation process, by becoming opaque and brittle.

### 2.3. Characterization Methods

A LUMOS Microscope Fourier Transform Infrared (FTIR) spectrophotometer (Bruker Optik GmbH, Ettlingen, Germany), equipped with an Attenuated Total Reflection (ATR) device. ATR-FTIR was used to acquire spectra in the range 600–4000 cm^−1^.

The scanning electron micrographs of PLLA samples were registered with a Quanta 200 microscope at an accelerating voltage of 15 kV and with an Energy Dispersive Spectroscopy (EDAX) system of elemental analysis (FEI Company, Brno, Czech Republic).

Transmission electron microscopy (TEM) images of CS/Ag Np solution were obtained on a HT1700 (Hitachi High-Technologies Corporation - Hitachi High-Tech, Tokyo, Japan) microscope using an acceleration voltage of 120 kV.

A Nanostar U-system (Bruker AXS GmbH, Karlsruhe, Germany) equipped with a Vantec detector and an X-ray micro source was used to perform small-angle X-ray scattering measurements (SAXS). The sample-to-detector distance was 107 cm and the wavelength of the incident X-ray beam was λ = 1.54 Å (Cu Kα). The solutions were loaded in capillary tubes and together with the film samples were measured under vacuum at a constant temperature, 25 °C for 10,000 s. Data analysis was performed by the model fitting approach using the DIFFRAC*^plus^* NanoFit.

Particle sizes from Cs/AgNp solution were measured using a Zetasizer instrument (Zetasizer Nano ZS, Malvern Instruments Ltd, Malvern, Worcestershire, UK) using UV (ultra violet) Grade cuvette after treatment in an ultrasonic water bath (Model FB11012, Fisherbrand, Loughborough, UK) for 30 min to break up any aggregates present. All measurements were performed in triplicate.

A sample of the CS/AgNP solution was diluted with distilled water (1:10 sample:water) and analyzed by spectrophotometry Ultraviolet–visible (UV-vis) produced by Barloworld Scientific Ltd, Dunmow, Essex, UK.

Films roughness were determined using a Tencor Alpha-Step D-500 stylus profiler (KLA Tencor Corporation, Milpitas, CA, USA) both before and following the immersion in the Cs/AgNP for the samples at 1000 µm scan length, and 100 µm/s scan speed. The arithmetic average of the absolute values of the profile heights over the evaluation length Ra was measured by applying a stylus force of 2.3 mg, and a long-range cutoff filter of 25 µm.

Static contact angle measurements using the sessile-drop method were performed on a CAM-101 (KSV Instruments Ltd., Helsinki, Finland) system equipped with a video camera, liquid dispenser and drop-shape analysis software (KSV CAM Optical Contact Angle and Pendant Drop Surface Tension Software, version 3.99, KSV Instruments Ltd., Helsinki, Finland). Liquid drops (double distilled water or ethylene glycol) of ~1 μL were placed at room temperature, with a Hamilton syringe, on the polymer surface. For each drop 10 photos were recorded at an interval of 0.016 s. To obtain a statistical result, three different surface regions were selected for each liquid.

### 2.4. Culture Media Preparation and MTT Test

The samples were cut in fragments of 5 × 5 mm size and were decontaminated by immersion in a sterile solution of 70% ethyl alcohol for 20 min. Then the samples were rinsed three times in sterile PBS and were pre balanced in complete culture media for 24 h at 37 °C.

The MTT test was performed by a direct contact method, by using as test product the samples prepared as described above in 24-well culture plates populated with preosteoblasts MC3T3-E1 line, subclone 4. The initial cell population density was 1 × 10^4^ cells/well, in 0.5 mL α MEM. The sample contact with the cells was made after 48 h after culture initiation, to obtain a cell monolayer semi-confluent. One piece of material with the size of 5 × 5 mm was then placed in each well over the cell culture for 72 h, at 37 °C, humidity 95% and 5% CO_2_. Each sample was tested in triplicate material and the results were compared with the ones obtained for control samples, without testing material. The MTT test with 3-(4,5-dimethylthiazol-2-yl)-2,5-diphenyltetrazoliumbromid was carried out according to techniques from the literature [27,28]. The principle of the method is based on the reduction of yellow MTT compound, in a violet-colored product (formazan), as a result of mitochondrial dehydrogenase activity of viable cells. To achieve the MTT assay, the culture medium and the fragment of the material from each well was removed, the cells MTT solution was added in α MEM without BFS. After 3 h of incubation solution, the formazan absorbance was measured using a spectrophotometer plate reader (Tecan) at a wavelength of 570 nm. By reporting formazan absorbance from the wells with experimental samples to the control ones, the percent cell viability corresponding to the incubated culture with one of the tested materials was calculated.

### 2.5. Antimicrobial Activity

The polymers were cut into squares (1 cm side), plated in 24-well plates, sterilized with 70% ethanol (1) and washed with PBS (3 times). The test polymers were then incubated with 1 mL of bacterial suspension (*S. aureus strains*, *P. aeruginosa*, 0.5 McFarland turbidity). The plates were placed in the incubator at 37 °C for 2 h. Thereafter, the polymers were removed from the 24-well plates using a sterile forceps and were washed three times with PBS to remove the non-adherent bacteria. The films were then placed in tubes with 1 mL of PBS and vortexed for 120 s to remove all solutions from the adhering bacteria. Then, the solution was serially diluted in PBS, cultured on nutrient agar and the number of colonies forming units per ml (UFC/mL) was calculated.

## 3. Results and Discussions

In this work, chitosan was used as a mild reducing agent in silver nanoparticle synthesis. Protonized chitosan with NH_3_^+^ functional groups was obtained by reaction of chitosan with H^+^ from the acetic acid solution. At the same time, the positions of Ag^+^ were fixed by coordination to the functional groups of chitosan, which simultaneously act as a stabilizing agent. The biopolymer behaves as a template or matrix which prevents the nanoparticles agglomeration.

The effect of surface modification experiments can be permanent (in the case of covalent attachment of functional groups) or non-permanent (non-covalent attachment). In the case of PLLA plasma treatment, the advantage is due to the improvement of surface wettability and cell affinity, while the disadvantage is due to surface rearrangement to minimize the interfacial energy, which affects the effectiveness of the surface modification, making the effect of plasma treatment non-permanent [29,30]. Another disadvantage of plasma treatment is degradation of PLLA in certain conditions. The two-step method described in our paper overcomes those disadvantages by immersing the PLLA plasma treated film in the chitosan-based AgNp solution immediately after the treatment, without any delay. In this way, the surface rearrangement does not have time to take place and the newly-introduced functional groups are efficient in permanent attachment of AgNP to the treated polymer surface. On the other hand, the input plasma parameters (the treatment time) were tailored to obtain no degradation of the PLLA film, the samples obtained in optimal conditions being selected to be presented in this paper. At lower values of the plasma treatment, no important changes in the film surface were noticed, while for higher values of the treatment, the PLLA film becomes brittle and opaque, the bulk proprieties being seriously affected.

**Figure 2:**
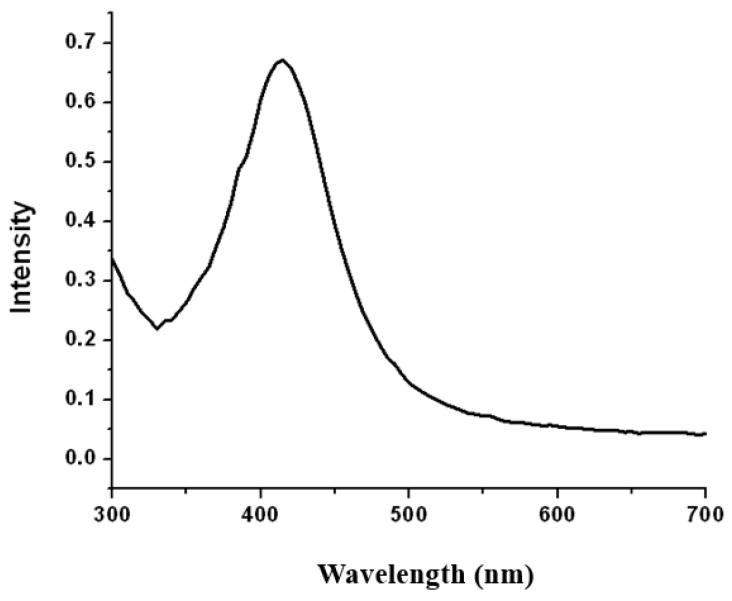
Ultraviolet–visible (UV-vis) spectrum of CS/AgNp solution.

The synthesis of AgNp in CS was demonstrated by UV–vis spectroscopy. The concentration of the chitosan-capped silver nanoparticles was approximately 0.11 ng/mL. The presence of surface plasmon resonance in the UV–vis characteristic optical spectrum indicates the presence of silver nanoparticles of certain particle size [31]. The UV-vis results (Figure 2) show a typical silver absorption peak at 415 nm which is in the reported range of silver and silver oxide nanoparticles [32,33,34]. The symmetry of the nanoparticles can be determined by the number of surface plasmon resonance peaks. If only one peak is observed in the UV-vis spectra, spherical silver nanoparticles were synthesized. The absorption peak is relatively narrow; thus, this method revealed a small size distribution of the particles in solution and the absence of unreduced positively charged ions, which is consistent with the data observed in the SEM and TEM images.

### 3.1. TEM Images and Particle Size Results of CS/AgNp 

**Figure 3:**
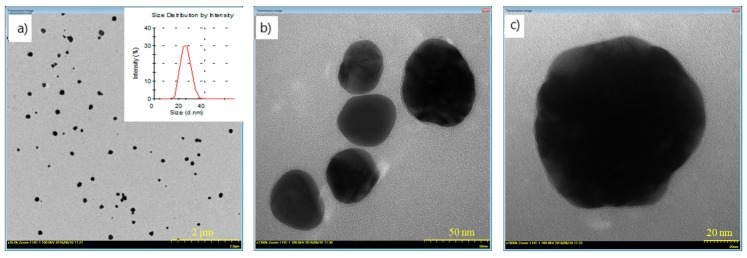
Transmission electron microscopy (TEM) images of CS/AgNp solution and Ag particle size distribution for scale: (**a**) 2 µm; (**b**) 50 nm; (**c**) 20 nm.

The representative TEM images of AgNPs show that the particles were randomly distributed in the solution and due to the presence of the polymer, no agglomeration was detected (Figure 3a). The particles in Cs/Ag Np solution have a spherical shape (Figure 3b) and an average particle size of about 30 nm (Figure 3c) was obtained, in good concordance with the particle distribution obtained by dynamic light scattering method (Figure 3a). Other authors obtained similar results [35]. The contrast of TEM micrographs is correlated with the nature and size of the particles. The main discussion is between organic and inorganic particles or different organic-inorganic composites. Soft materials are predominantly composed of low number atoms, such as C, O and N. These elements, compared to heavy metals, exhibit a low level of electron-optical contrast. Thus, TEM micrographs of CS/AgNPs show AgNP formation—which appears as dark areas due to the high electron density of Ag. The contrast difference for CS/AgNP is because by drying the nanoparticle suspension, chitosan remains at the surface of the particles, and thus gives rise to areas of low contrast compared to AgNPs.

### 3.2. SAXS Results

**Figure 4:**
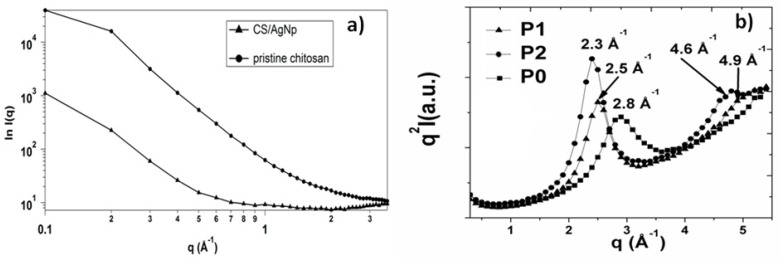
SAXS patterns: (**a**) double-logarithmic plot for pristine chitosan and CS/AgNp solution; (**b**) Kratky plot for pristine and treated PLLA samples.

The confirmation of particle size as obtained from TEM images was further authenticated by the SAXS analysis performed for all studied samples and the results are illustrated in Figure 4. The SAXS plots on a double logarithmic scale of the pristine chitosan and CS/AgNp solution presented in Figure 4a) demonstrate no important modification in chitosan morphology after the reduction of silver. The change in the slope of the CS/AgNP SAXS pattern is due to the presence of the silver nanoparticles in the system, and by applying the spherical model using DIFFRAC*^plus^* NanoFit, a value of 35 nm was obtained, in good concordance with TEM results.

The structural changes in the PLLA samples after applying the two-step treatment is demonstrated by the Kratky Plot obtained from SAXS measurements (Figure 4b). It is well known that plasma treatments are accompanied by the heating of the polymer due to the interaction with high-energy plasma particles with the material surface. On the other hand, the crystal modification of PLLA is easily obtained from the melt [36]; therefore, the increase in the intensity and the shift at smaller angles of the PLLA peak in the Kratky plots is a clear indication that the process of PLLA crystallization takes place (Figure 4b). The appearance of the second SAXS peak as the treatment time in plasma increased suggests the formation of regular aligned lamellar structures in the polymer matrix due to the plasma treatment observed by other authors [37]. The average long period of a lamella L can be estimated from the maximum of the peak (q_max_) in the Kratky plots (Figure 4b) according to Bragg’s law (L = 2π/q_max_). The thickness increased with the increase of plasma treatment time and lamellar structures grew in size from 0.25 nm for P1 to 0.27 nm to P2.

### 3.3. FTIR Results

FTIR measurements were carried out to elucidate the interactions that take place in the reduction process of silver nitrate in the presence of chitosan.

**Figure 5:**
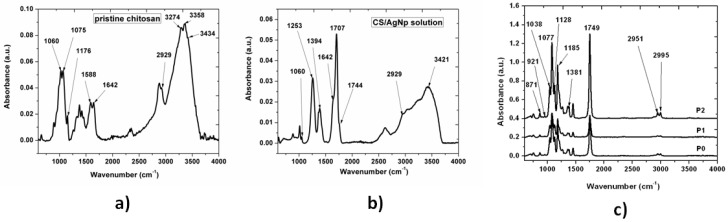
Fourier Transform Infrared (FTIR) results for (**a**) pristine chitosan; (**b**) CS/AgNp solution; (**c**) pristine and treated PLLA samples.

In the pristine chitosan spectrum from Figure 5a, the broad absorption peak in the 2250–3800 cm^−1^ region is attributed to symmetric and asymmetric vibrations of CH_2_ (2250–3050 cm^−1^), and vibrations of O–H, N–H and intermolecular hydrogen bonds of polysaccharides (3050–3800 cm^−1^). The peaks at 1588 and 1642 cm^−1^ were assigned to amino (–NH_2_), amide I (C=O) and C=O of O–C–O–R groups in the chitosan structure, respectively. The peaks at 1060, 1075 and 1176 cm^−1^ are the characteristic absorptions due to C–O vibrations in the C–O–C band [38]. The formation of silver/chitosan nanoparticles was confirmed by Fourier transform infrared spectroscopy. As shown in Figure 5b, the spectra of the CS/AgNP exhibited a few differences from the chitosan. The peak intensities in the range 1000 cm^−1^ and 1350 cm^−1^ due to C–N stretching and bending decreased because of the reduction of silver in chitosan. The absence of 1588 cm^−1^ peak that exists in pristine chitosan and the appearance of additional peaks at 1707 and 1744 cm^−1^ (Figure 5b), corresponding to carbonyl stretch vibrations in ketones, aldehydes and carboxylic, indicate that the silver is bound to the functional groups of chitosan. The formation of chitosan- silver nanoparticles was achieved after the reduction of silver ion through the amino group. The presence of these functional groups on the surface of the synthesized silver nanoparticles and the disappearance of the NH_2_ double spike peak indicates that the polymer successfully capped the nanoparticles and the polymer network restricts the diffusion of Ag^+^. Moreover, the reduction of the silver ions is coupled to the oxidation of the hydroxyl groups in chitosan molecular and/or its hydrolyzates acids [39]. The band at 3434 cm^−1^ assigned to the overlap between the O–H stretching vibration and the N–H stretching vibration of the biopolymer moieties, shifted to 3421 cm^−1^ due to co-ordination bond between the silver and electron rich groups [40,41]. After silver binding during reduction of silver nitrate with chitosan, the molecule weight was heavier and the vibration intensity of the N-H bond decreased, suggesting the attachment of silver to nitrogen atoms from chitosan [42].

Figure 5c shows the IR spectra of the PLLA samples. The 1185 and 1077 cm^−1^ bands were assigned to C–O–C asymmetric and symmetric stretching, while the peak at 1749 cm^−1^ was attributed to the stretching of C=O. The C–CH_3_ stretching caused the peak at 1038 cm^−1^ and the C–H (of CH_3_ groups) rocking mode was present in the spectrum at 1128 cm^−1^. An increase in the degree of PLLA crystallinity due to the heating of the polymer during the plasma treatments can be observed, which is in good concordance with SAXS measurements. There was an increase of the 1749 cm^−1^ and of 1381 cm^−1^ band intensities. A shift with 3 cm^−1^ to lower wavenumbers of those bands can be observed. Those bands were assigned to the carboxylic groups and demonstrated an increase of those functional groups quantity after plasma treatments and the presence of silver ions at the polymer surface [43,44]. There was an increase of the 1749 cm^−1^ and of 1381 cm^−1^ band intensities assigned to the carboxylic groups, which demonstrate an increase of those functional groups quantity after plasma treatments and the presence of silver ions at the polymer surface. A shift with 3 cm^−1^ to lower wavenumbers of those bands can be observed, which revealed the reaction between the carboxyl groups in PLLA and the amino groups in CS; PLLA was grafted onto the backbone of CS.

### 3.4. Surface Roughness and Wettability

The polymer surface was affected by the plasma treatment due to the breaking of chemical bonds, heating, degradation, etc. [26]. Because all of these processes may significantly change the structure and morphology of the polymer film, the surface roughness and contact angle measurements were performed.

Hydrophobicity and hydrophilicity proprieties of polymer surface are key factors in further cell adhesion. Surface functional groups and the surface roughness of the material are very important in determining surface wettability.

To determine the wettability of neat and treated PLLA surfaces, water contact angle measurement (two liquids method) was used to provide the information on the wetting properties (Table 1). The values of the static contact angle (θ_w_ for water and θ_EG_ for ethylene glycol) can be used to estimate the wettability and surface tension of a solid surface. Based on these measurements, some parameters such as surface free energy (γ_SV_), solid–liquid interfacial tension (γ_SL_), or work of adhesion (W) were calculated using Owens–Wendt–Rabel and Kaelbe methods [45,46,47] and the results are listed in Table 1. The polar and the dispersive components of surface free energy were also listed in Table 1, to evaluate the surface modifications after the two step treatment.

The roughness parameters measured with profiler revealed that the increase of treatment time causes an increase of the surface roughness parameters (Table 1).

**Figure 6:**
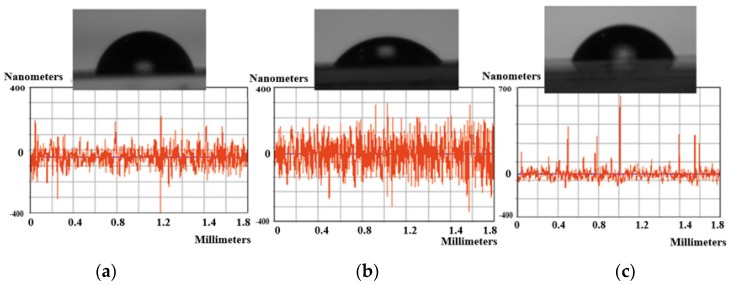
Surface modification results: top figures—contact angle images; bottom figures—profiler roughness measurements of samples: (**a**) P0; (**b**) P1; (**c**) P2.

Both treatments caused a significant increase in the surface roughness and a decrease in the static water contact angle compared to the untreated polymer. The lowest water contact angles and the highest value for surface roughness were obtained for P1 treatment (Figure 6).

The values listed in Table 1 revealed that both plasma treatments significantly increased the surface energy, mainly due to the increase in its polar component. Furthermore, the lowest value of the water contact angle was achieved at the largest values of the polar component of the surface energy.

### 3.5. SEM and EDAX Results

**Figure 7:**
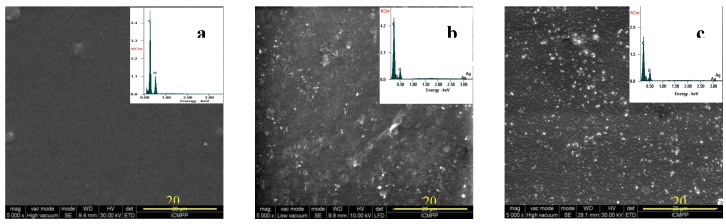
SEM images and Energy Dispersive Spectroscopy (EDAX) spectra of: (**a**) P0; (**b**) P1; (**c**) P2.

The morphological changes in the PLLA surface after the two-step treatments are presented in Figure 7. From Figure 7a, pristine PLLA film has a smooth surface without any irregularities. The PLLA films surfaces, after the combined plasma-wet chemical treatment, have patterns of different size and shape due to the surface interactions with different reactive species formed in plasma and due to the presence of AgNp and chitosan (Figure 7b,c, respectively). EDAX measurements (Table 2) show the presence of the AgNp on the surfaces of the P1 and P2 films after the treatments. The presence of nitrogen in the treated samples supports the presence of chitosan at the polymer surface.

It can also be observed that the highest amount of nitrogen was obtained in the P1 sample, while the high amount of Ag was obtained in the P2 sample. The carbon concentration was slightly increased with the increase of the plasma treatment time as a result of the polymer chain destruction. The low concentration of Ag nanoparticles assures low toxicity at the surface of the polymer and avoids the agglomeration of nanoparticles. Moreover, the presence of N at the treated samples surfaces demonstrates the presence of chitosan.

P1 and P2 samples were treated in plasma at different conditions and then introduced in the same solution of CS/AgNp. After plasma treatment, functional groups of different concentrations (depending on the input plasma parameters) were present at the PLLA surface. Those groups are responsible for the presence of chitosan and silver at the PLLA treated surfaces, as presented in FTIR section. From Table 2, the modified N/C ratio on the surface was 0.19 for P1 and 0.17 for P2, while Ag/C ratio was 0.006 for P1 and 0.011 for P2. A possible mechanism responsible for the higher amount of Ag and the smaller amount of N in P2 sample compared to P1 is: unreduced silver from CS/AgNp solution can easily direct bond to the PLLA surface containing functional groups, without the aid of chitosan. On the other hand, if the larger chitosan molecule does not find sufficient functional groups with which to form bonds at the PLLA surface, it is removed by washing after treatment.

### 3.6. Proliferation and Morphology of on the MC3T3-E1 Cells on the PLLA Samples

Clinical biomaterial applications require good biocompatibility of the material. The biocompatibility of neat PLLA and two step treated PLLA films can be primarily evaluated by utilizing MC3T3-E1 cell lines, to be used for applications such as bone tissue engineering. 

**Figure 8:**
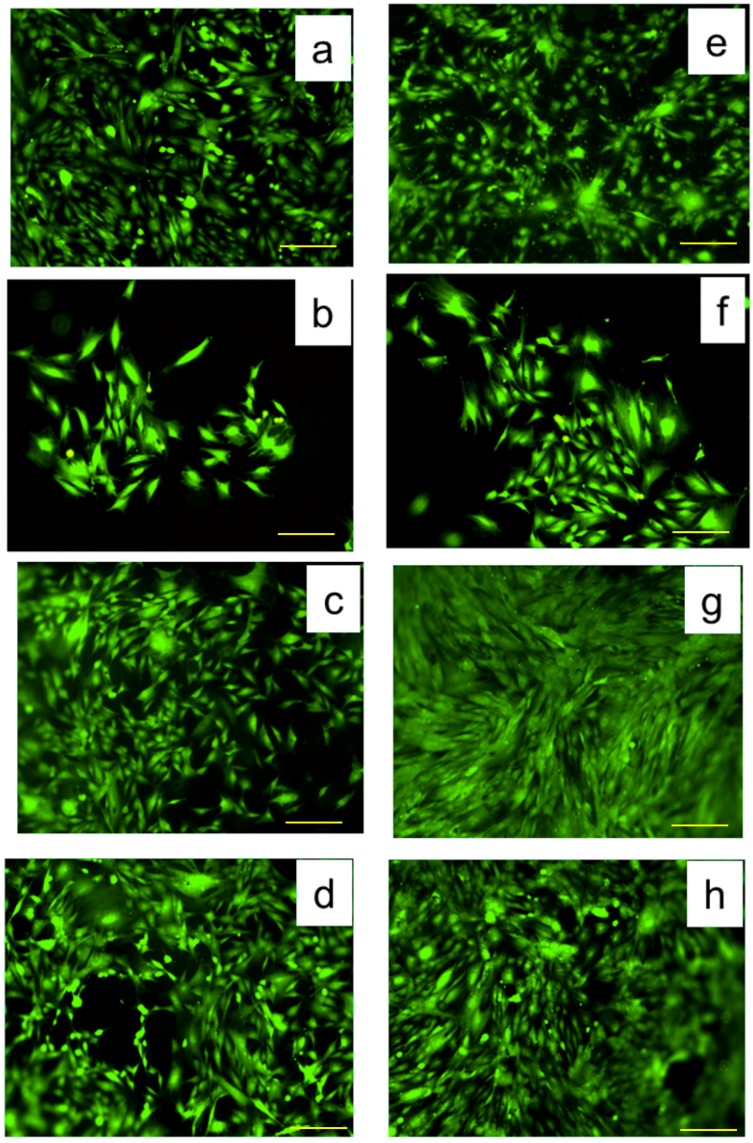
Cell culture (cell line MC3T3-E1) after 48 h for (**a**–**d**) and after 72 h (**e**–**h**); control (**a**,**e**), P0 (**b**,**f**), P1 (**c**,**g**), P2 (**d**,**h**). Scale: 200 µm.

Fluorescent staining was used to study cell density and morphology after culturing MC3T3-E1 cells on P0, P1 and P2 films for 48 h and 72 h, as shown in Figure 8. For plasma—treated samples P2 and P0, spherical and round cells can be observed. The growth of cells on the surface of P1 films was better and more rapid than that on the P0 and P2 samples (Figure 8h) after culturing for 72 h. Moreover, the cells incubated on P1 show a higher degree of fibroblast cell adhesion and proliferation and a well-preserved morphology, which was flat and fully spread.

As a measure of unsaturated bond energy resulting from dangling bonds of surface material [42,43] surface energy is an important chemical cue on polymer surfaces. A polymer in contact with biological fluids has a surface energy which influences cell activities, such as serum protein adsorption and cell attachment. It was found that more fibroblasts can adhere and spread widely on the more hydrophilic polymer surface. The improvement or the suppression of cell adhesion at the polymer surface is in good concordance with high or respectively low surface free energy values [44,45]. In the case of equal surface free energy, a higher value for the polar component will induce a higher degree of cell adhesion and proliferation on the surface. The surface energy of the PLLA surface was tailored by using plasma treatment and the surface free energy and the polar component of the P1 film was higher (Table 1) than that of the P0 and P2 films.

Two different cell morphologies were found on the studied polymer surfaces (Figure 8): (1) elongated cells well spread into polymer surface and (2) rounded cells, which are attached but have not begun to spread. Both types of morphologies are seen in different proportions on studied samples. The majority (>80%) of cells grown on PLLA films are elongated. On each of the P0, P1 and P2 substrates, up to 20% of cells were rounded. The highest density of elongated cells was found at P1 treatment after 72 h.

**Figure 9:**
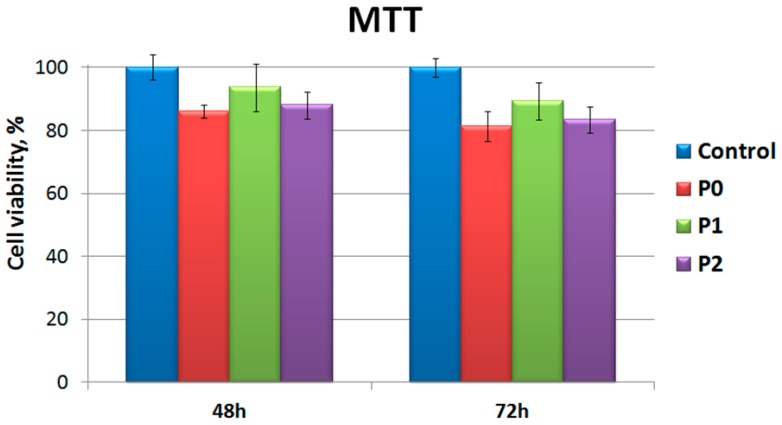
MTT assay results of MC3T3-E1 cells on the P0, P1 and P2.

Figure 9 shows the MTT assay results of the MC3T3-E1 cell lines on P0, P1 and P2 films on 48 h and 72 h. The number of cells in each group increased with culture time on all of the tested groups. The MC3T3-E1 cells cultured on all samples have similar proliferation on the first day compared with the control. From the first day, the viable cell numbers on P1 were higher than those on P0 and P2.

The PLLA/Chitosan based-silver nanocomposite scaffolds appeared to be in vitro biocompatible and noncytotoxic to cells (Figure 9). The higher density of cells on P1 samples can be attributed to a higher concentration of N and a lower Ag concentration at film surfaces (Table 2 EDAX measurements). The shape, dimensions and low concentration of the silver ions prove to be nontoxic for the MC3T3-E1 cells culture. Moreover, the highest concentration of N and the highest polar component obtained for P1 samples assures better biocompatibility of P1 samples even compared to the neat PLLA.

### 3.7. Antimicrobial Activity

**Figure 10:**
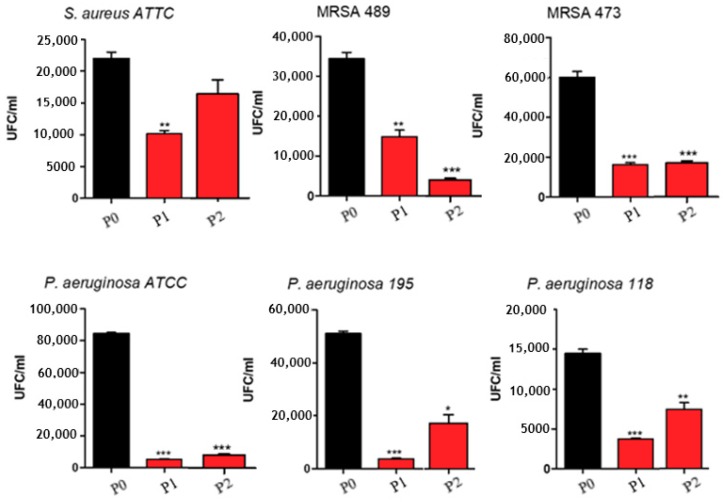
Strains adherence of *S. aureus* and *P. aeruginosa* (ATCC, clinical isolates) to polylactic acid films; *, ** and *** correspond to the magnitude of antimicrobial activity.

Strains adherence of *S. aureus* and *P. aeruginosa* (ATCC, clinical isolates) to polylactic acid films were studied. Figure 10 demonstrates the very good antimicrobial proprieties of P1 and P2 samples compared to the untreated P0 sample. The antimicrobial behavior of P1 sample is more pronounced in the case of *P. aeruginosa* compared to *S. aureus* and can be explained considering that both chitosan and AgNp have a bactericidal effect.

One of the proposed mechanisms of Ag action was based on AgNp capability to easily enter into the bacterial cell and form a less dense region in the center of the bacteria, causing the cell death by interacting with thiol containing enzymes [46,47]. Another proposed mechanism involves disruption of DNA/RNA caused by Ag reaction with the weak acid groups in the genetic material, such as phosphate [48].

On the other hand, chitosan has antimicrobial behavior and the main mechanism is based on the electrostatic interaction between positively charged chitosan groups and negatively charged sites on microbial cell [49]. In concordance with other authors [50], Figure 10 demonstrated that chitosan has stronger influence on Gram negative than on Gram-positive strains because the cell wall of *P. aeruginosa* (Gram-negative) has a thickness of 7–8 nm while the wall of *S. aureus* (Gram-positive) is around 20–80 nm [51].

## 4. Conclusions

In this paper, an environmentally-friendly synthesis of metallic nanoparticles in the presence of chitosan was performed. Silver ions underwent coordination and reduction thanks to the presence of numerous amino and hydroxyl groups in the chitosan chains. Bounding of silver nanoparticles of 30 nm average diameter to the polymer functional groups ensured a long-term stability and prevented their agglomeration. FTIR data pointed out the possible interactions of the hydroxyl or amino groups of chitosan and the carboxyl groups of PLLA. The silver nanoparticles were successfully adsorbed on PLLA films exposed to plasma treatments, by simply immersing the treated films in the chitosan solution containing silver nanoparticles. In this way, chitosan was used to fix silver nanoparticles on PLLA films surfaces. This is a time saving, inexpensive and eco-friendly synthesis that minimizes the use of toxic chemicals and does not produce toxic waste. 

The biopolymer-based nanocomposite scaffolds with bioactive inorganic phases are of high interest due to their biocompatibility in combination with preosteoblastic cell line MC3T3-E1 (established from mouse C57BL/6 calvaria) seeding. The sample, which has demonstrated from EDAX to have the highest amount of nitrogen and the smallest amount of Ag, proved to have the highest value for cell viability. Moreover, it demonstrated better biocompatibility and very good antimicrobial activity against gram-negative and gram-positive bacteria. The effective component for the biocompatibility seemed to be both PLLA and chitosan, while for the antimicrobial property both chitosan and silver were responsible.

The described two-step method is a promising technology for obtaining: poly(L-lactic acid) for tissue engineering applications like bone regeneration. In this direction, the new approach of biopolymer-polysaccharides based composite enables the scaffold surface to mimic complex local biological functions. To target clinical and medical applications, the need for additional investigations in the biological system is imperative.

## Figures and Tables

**Table 1:** Surface roughness and wettability results.

	Roughness	Contact Angle Measurements Parameters							
	Ra (nm)	θ_w_	θ_EG_	W_w_	W_EG_	γ_SV_^p^	γ_SV_^d^	γ_SV_	γ_SL_
P0	43.103	79.9	52.19	86.19	77.42	8.05	23.93	31.98	18.58
P1	86.448	51.54	34	118.07	87.79	38.93	9.61	48.55	3.27
P2	63.831	70.11	42.22	97.56	83.54	13.96	22.39	36.36	11.60

**Table 2:** EDAX results of PLLA studied samples.

Element (%)	CK	OK	NK	AgL
P0	70.49	29.51	-	-
P1	71.73	14.36	13.46	00.45
P2	72.43	14.61	12.14	00.82
